# Sub‐Acromioclavicular Decompression Increases the Risk of Postoperative Shoulder Stiffness after Arthroscopic Rotator Cuff Repair

**DOI:** 10.1111/os.14225

**Published:** 2024-09-28

**Authors:** Cheng Li, Zhiling Wang, Maslah Idiris Ali, Yi Long, Ymuhanmode Alike, Min Zhou, Dedong Cui, Zhenze Zheng, Ke Meng, Jingyi Hou, Rui Yang

**Affiliations:** ^1^ Department of Orthopedics, Sun Yat‐sen Memorial Hospital Sun Yat‐sen University Guangzhou P. R. China

**Keywords:** Postoperative Shoulder Stiffness, Rotator Cuff Tears, Sub‐acromioclavicular Decompression

## Abstract

**Objective:**

The sub‐acromioclavicular (SAC) decompression is often performed during arthroscopic rotator cuff repair. However, the impact of SAC decompression on patients with postoperative shoulder stiffness (POSS) are controversial and unclear. This study is aim to evaluate the impact of additional sub‐acromioclavicular (SAC) decompression during arthroscopic rotator cuff repair on the postoperative shoulder stiffness (POSS) in patients.

**Methods:**

This retrospective study examined digital data from patients with full‐thickness rotator cuff tears who underwent arthroscopic rotator cuff repair at a local institution. Patient‐reported outcomes were evaluated using the American Shoulder and Elbow Surgeons (ASES) Score, the University of California–Los Angeles (UCLA) score, and visual analog scale (VAS) scores. Restricted shoulder mobility occurring within 6 months postoperatively, lasting more than 12 weeks, characterized by a passive forward flexion angle of <120° or an external rotation angle of <30°, with or without associated shoulder pain was identified as POSS. Factors affecting POSS were analyzed by binary logistic regression analysis. The patient‐reported outcomes scores were analyzed by generalized estimating equations to examine the impact of SAC decompression.

**Results:**

A total of 155 patients met the set criteria and were included in the study. The analysis of binary logistic regression showed that diabetes (*p* = 0.001) and SAC decompression (*p* = 0.003) were independent factors for POSS. In the analysis of each follow‐up point, only at the 3‐month follow‐up, the ASES scores (*p* = 0.003), UCLA scores (*p* = 0.045), and VAS scores (*p* = 0.005) showed significant differences between the SAC decompression group and the non‐decompression group. For the intergroup comparison, the results showed a significant difference in the ASES scores (β = −4.971, *p* = 0.008), UCLA scores (β = −1.524, *p* = 0.019), and VAS scores (β = 0.654, *p* = 0.010) throughout the study duration between the SAC decompression group and the non‐decompression group.

**Conclusion:**

The findings of this study suggested that SAC decompression during arthroscopic rotator cuff repair increase the risk of POSS compared with those without the decompression, which indicate surgeons do not perform SAC decompression unless necessary.

## Graphical abstract

SAC decompression increases the risk of POSS compared with the normal group after arthroscopic rotator cuff repair. However, orthopedic surgeons should consider the individual clinical data and goals of each patient when making decisions about surgical procedures.

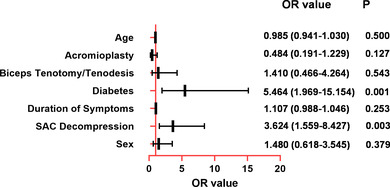

## Introduction

Rotator cuff tear is the prevalent shoulder disorder that leads to shoulder dysfunction and pain experience for many patients.^1^, ^2^ Arthroscopic rotator cuff repair is a widely used treatment option for this condition due to its rapid recovery of shoulder function and the ability to quickly return to normal activities.^3^, ^4^, ^5^ Despite its benefits over other therapies, numerous studies have reported the postoperative complications, such as postoperative shoulder stiffness (POSS), infection, and deep vein thrombosis and others.^6^, ^7^, ^8^ Researches have shown that POSS could be found up to 11%–31% in the 6 months following‐up after surgery, leading to prolonged pain and rehabilitation and, in some severe cases, additional treatment including intra‐articular injections and surgery.^9^, ^10^, ^11^, ^12^, ^13^

Postoperative shoulder stiffness refers to a reduction in the passive range of motion (ROM) of the shoulder joint that surpasses normal postoperative pain or stiffness, making it difficult for patients to perform daily activities and exercise.^8^, ^9^, ^14^ This condition can cause pain, weakness, and decreased function in the affected shoulder. POSS after surgery may be caused by the adhesions and scarring within the joint and soft tissue contracture.^8^ There are several clinical factors in developing POSS, including age, sex, the size and location of the tears, preoperative stiffness, surgical technique, and rehabilitation protocol.^15^, ^16^, ^17^, ^18^ However, while other clinical factors have been shown to have an impact on POSS, only a limited number of studies that have evaluated the influence of surgical procedures on POSS after surgery.

Sub‐acromioclavicular decompression (SAC) is a frequent surgical procedure in arthroscopic rotator cuff repair that aims to decrease impingement and abrasion for the supraspinatus tendon in the shoulder joint.^19^, ^20^ This is typically accomplished by removing bony spurs and soft tissue around the tendons that are causing the impingement. Some studies had reported that sub‐acromioclavicular (SAC) decompression is performed simultaneously with arthroscopic rotator cuff repair to improve the surgical outcome.^8^, ^19^, ^21^ However, the surgical procedure itself can cause damage to the soft tissue and joint capsule, potentially leading to increased stiffness. The relationship between SAC decompression and the development of POSS is not well understood and requires further clinical investigation.

This study is aim to (i) identified the risk factors of POSS in patients with full‐thickness rotator cuff tears, (ii) assess the impact of the additional SAC decompression during arthroscopic rotator cuff repair on POSS. We hypothesize that SAC decompression during arthroscopic rotator cuff repair can increase the risk of POSS compared with those without the decompression.

## Materials and Methods

The present study was performed by reviewing medical data obtained from a local digital shoulder platform. This registry‐based study, which aimed to research clinical data and surgery records, was approved by the ethics committee of the Sun Yat‐sen memorial hospital (SYSKY‐2023‐449‐01).

Patients who received a preoperative MRI diagnosis of full‐thickness rotator cuff tears between January 2019 and January 2022 and underwent arthroscopic rotator cuff repair were included in the study. Inclusion criteria included: (i) aged 40 years or older; (ii) confirmed full‐thickness tear through MRI; (iii) received arthroscopic rotator cuff repair in our institution; and (iv) have completed regular follow‐up questionnaires. Exclusion criteria included: (i) irreparable tear; (ii) scapula fracture or tumor; (iii) revision surgery; and (iv) other conditions that would prevent surgical management. The surgical procedures were carried out at our hospital by a single experienced shoulder surgeon.

Patient characteristics including age, sex, body mass index (BMI), dominance of affected shoulder, duration of symptoms, and diabetes mellitus. Physical examinations and patient‐reported questionnaires were completed for all participants. The definition of POSS is restricted shoulder mobility occurring within 6 months postoperatively, lasting more than 12 weeks, characterized by a passive forward flexion angle of <120° or an external rotation angle of <30°, with or without associated shoulder pain.

The patient‐reported outcomes were assessed by American Shoulder and Elbow Surgeons (ASES) scores, the University of California, Los Angeles (UCLA) score, the pain visual analog scale (VAS) scores, which are comprehensive and well characterized and accepted in the scientific community.^22^ The questionnaire is administered by doctors from the Department of Sports Medicine in our clinic, who interview patients and record the conclusions. The maximum range of flexion is defined as the maximum extent to which the upper limb can be lifted along the sagittal plane to the front of the body. Flexion strength is measured when the patient flex both upper limbs to 90 degrees and exert force against the doctor. The doctor records the muscle strength of the affected limb according to the Manual Muscle Testing (MMT) grading system. The surgical records included Operation time, number of anchors, tear size, tear muscles, biceps tenotomy/tenodesis, and acromioplasty. Patients were regularly evaluated at preoperative time, 3‐month following‐up point, and 6‐month following‐up point after surgery with the documentation of physical examinations and patient‐reported outcomes as previous research.^9^

### Surgical Procedures

**FIGURE 1:**
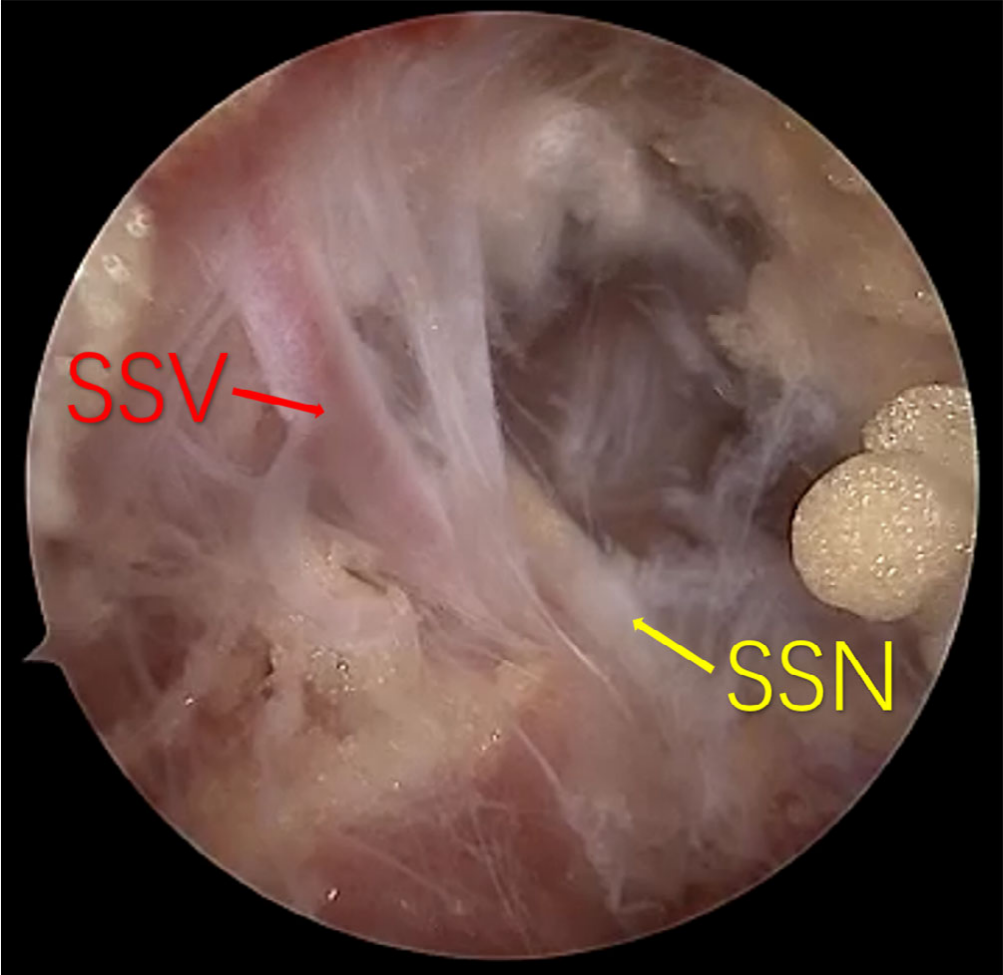
Arthroscopic view using 30° arthroscope from the lateral portal after SAC. The SSA, suprascapular artery (red) and SSN, suprascapular nerve (yellow) are showed in subacromial space.

All arthroscopic rotator cuff repair procedures were performed by one professional shoulder surgeon with patients in the lateral decubitus position or beach‐chair position under general anesthesia combination with brachial plexus block. The affected shoulder was positioned in 20 degrees of forward flexion and 35–45 degrees of abduction during the procedure. Continuous traction was used to the ipsilateral upper extremity to enlarge the operative space in the glenohumeral joint. After the standard operative portals were established, the diagnostic arthroscopy was performed to evaluate the full‐thickness tear. The selection of the repair type (double‐row or suture‐bridging double row) was based on the tear's extent and the mobility of the torn rotator cuff. Additionally, the frayed long head of the biceps was repaired through either tenotomy or tenodesis. The acromioplasty procedure was used for the patients with high critical shoulder angle or bony spurs of acromion. In the SAC depression group, SAC depression was performed by removing bursal tissue and soft tissue below the acromioclavicular joint using an arthroscopic soft tissue shaving resector. An arthroscopic motorized burr was then used to smooth the inferior surface of the acromioclavicular joint and remove any bony spurs or inferior osteophytes. Intraoperative pictures are showed in Figure 1. No SAC depression was performed in the non‐decompression group.

### Rehabilitation

**FIGURE 2:**
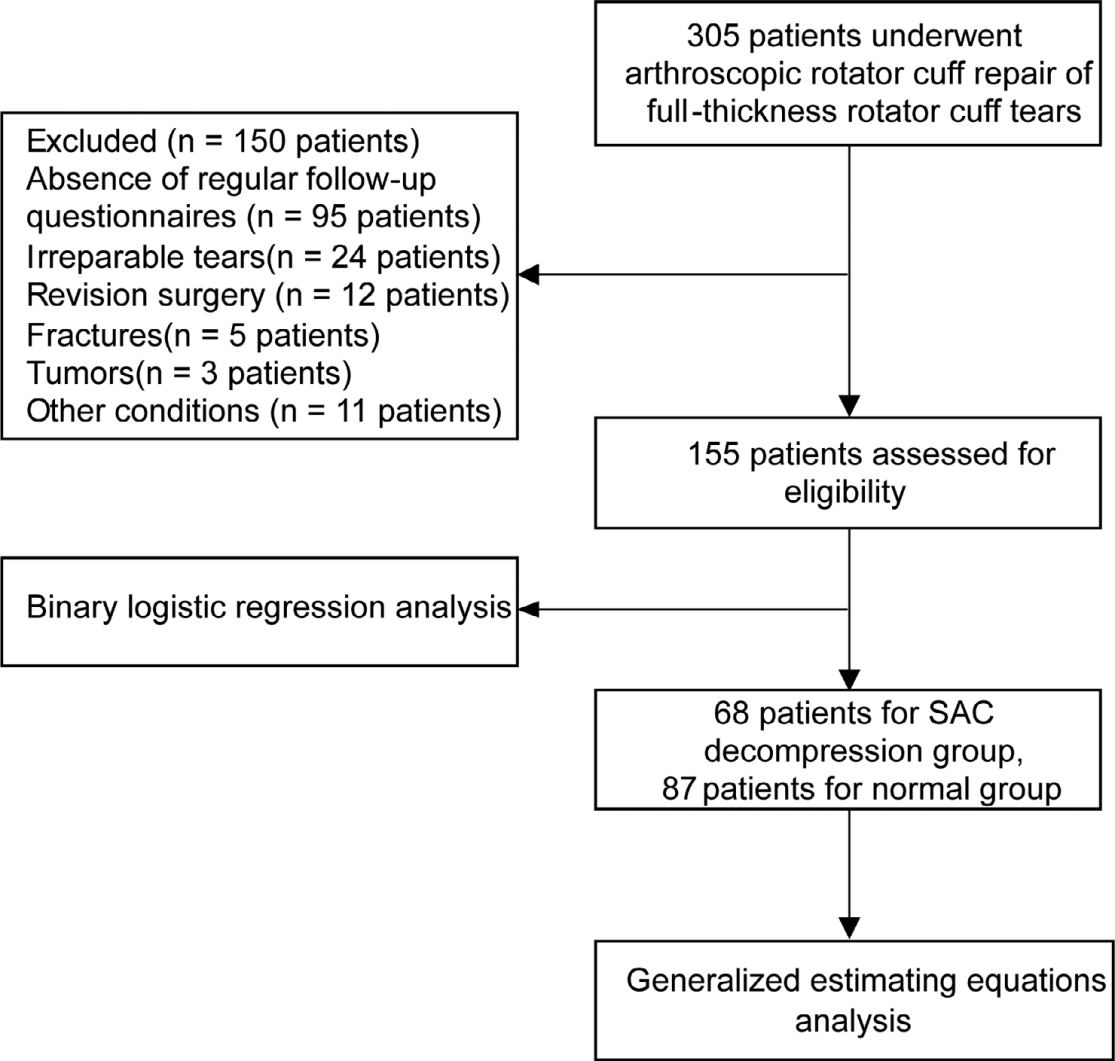
Flowchart of patient selection.

The following rehabilitation plan was implemented for all patients who underwent surgery.^23^ To begin with, patients received shoulder brace immobilization immediately after the surgery, followed by passive elbow and wrist activity on the 2nd day to maintain integrity of repair and minimize pain and inflammation. Starting from the 14th day, passive flexion of the shoulder was initiated to achieve staged range of motion (ROM) goals. After 6 weeks, active mobilization and coordination training of the elbow and shoulder commenced to promote healing of soft tissue and initiate light muscle performance activities and continued until 3 months post‐surgery. At last, patients would start progressive resistance exercises after 3 months and gradually return to the normal life. In addition, proper treatments with physical therapy, pain medication, and corticoid infiltration were be used for patients diagnosed as POSS (Figure 2).

### Statistics

The demographic data and patient‐report outcomes with continuous variables analyzed would be calculated as descriptive statistics, including means, and standard deviations (SD). The independent *t*‐tests were performed to compare the means of continuous variables between the SAC depression group and non‐decompression group. The clinical significance of categorical data was determined using the chi‐squared test. The variables were selected to binary logistic regression analysis by using the stepwise forward conditional method and regarding as important clinical factors. The generalized estimating equations were used were used to analyze the patient‐reported outcomes scores while controlling for important clinical factors such as sex, age, diabetes, Biceps tenotomy/tenodesis. A significance level of *p* < 0.05 was set. Statistical analyses were performed by using IBM SPSS Statistics (version 26.0; SPSS Inc., Chicago, IL, USA).

## Results

### Patients Selection

After conducting a retrospective evaluation of the digital database, a total of 305 patients with full‐thickness rotator cuff tears who underwent surgery were identified, and 155 of these patients were included based on the inclusion/exclusion criteria.

### Risk Factors for POSS

**FIGURE 3:**
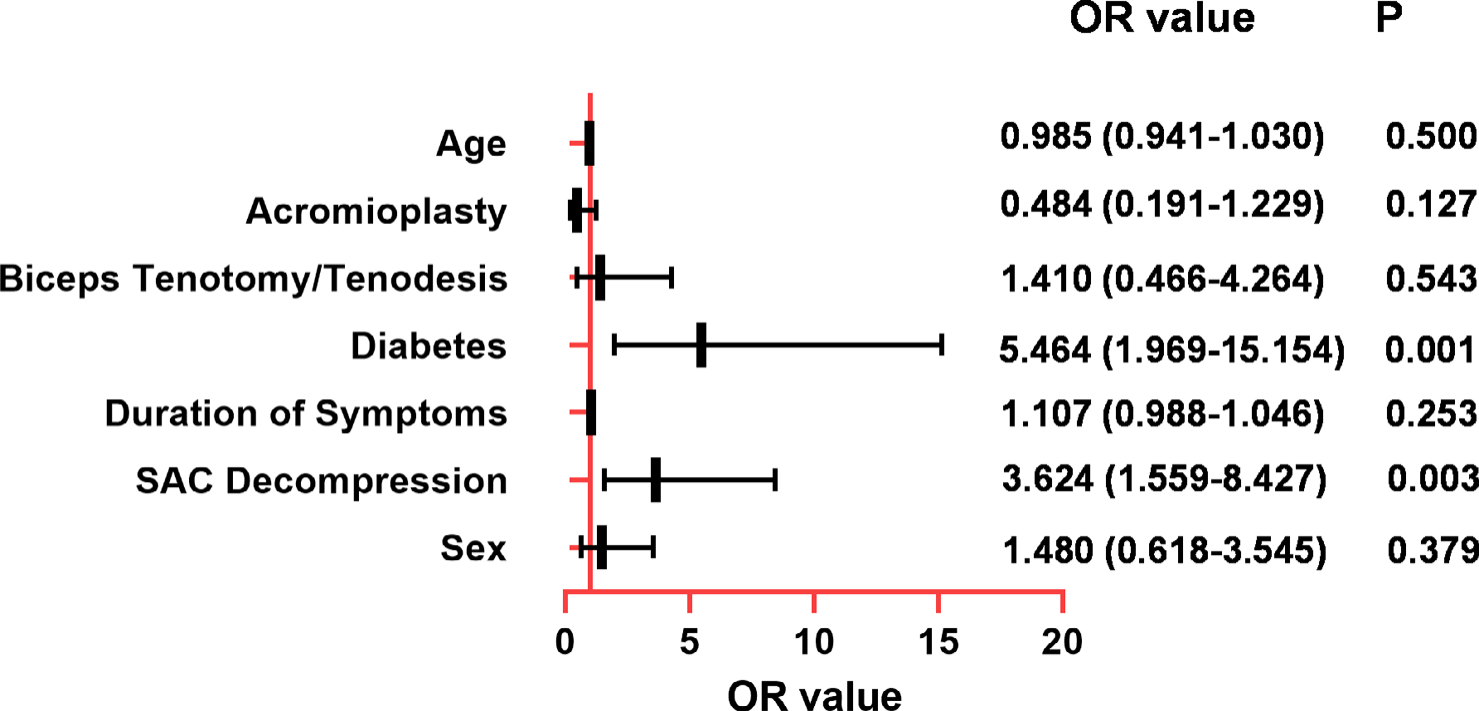
Results of binary logistic regression analysis. Among all included clinical characteristics, diabetes (OR = 5.464, 95% CI 1.969–15.156, *p* = 0.001) and SAC decompression (OR = 3.624, 95% CI 1.559–8.427, *p* = 0.003) were identified as independent risk factors for POSS.

On binary logistic regression analysis, the covariates, including sex, diabetes, dominant side, duration of symptoms, biceps tenotomy/tenodesis, acromioplasty, and SAC decompression were selected into analysis. The results showed that diabetes (OR = 5.464, 95% confidence interval (CI) 1.969–15.156, *p* = 0.001) and SAC decompression (OR = 3.624, 95% CI 1.559–8.427, *p* = 0.003) were independent factors for POSS. The results are shown in Figure 3.

### The Impact of SAC Decompression

Of all 155 patients, 68 patients were part of the SAC decompression group and 87 were part of the non‐decompression group. The patient characteristics are displayed in Table 1. The SAC decompression group had the significantly lower use of biceps tenotomy/tenodesis than the non‐decompression group (*p* = 0.017). However, there were no significant differences in other clinal factors between the two groups.

**TABLE 1:** Patient demographic data and operation records for SAC decompression group vs. non‐decompression group.

	SAC decompression group (*n* = 68)	Non‐decompression group (*n* = 87)	*p* value	Statistic value
Age (years)	60.53 ± 10.34	58.18 ± 9.49	0.144	1.467
Female, *n* (%)	41 (60.3)	60 (69.0)	0.215	2.712
BMI (kg/m^2^)	24.34 ± 3.15	23.63 ± 2.58	0.126	1.539
Dominant side, *n* (%)	52 (76.5)	73 (83.9)	0.245	1.353
Duration of symptoms (months)	11.96 ± 14.52	10.88 ± 10.95	0.601	0.524
Diabetes, *n* (%)	13 (19.1)	10 (11.5)	0.185	1.755
Operation time (minutes)	80.88 ± 26.67	85.94 ± 32.76	0.1305	−1.053
Number of anchors	3.76 ± 1.36	4.05 ± 1.28	0.190	−1.318
Tear size (mm)	21.63 ± 9.47	22.82 ± 11.40	0.508	−0.663
Tear muscles	1.93 ± 0.72	1.94 ± 0.80	0.897	−1.130
Biceps tenotomy/tenodesis, *n* (%)	49 (72.5)	76 (87.4)	0.017*	5.722
Acromioplasty, *n* (%)	47 (69.0)	67 (77.0)	0.269	1.222

Abbreviations: BMI, body mass index; SAC, sub‐acromioclavicular.

* Signifies statistical significance: *p* < 0.05.

There was significant difference in incidence of POSS between the two groups (*p* = 0.001) throughout the study duration. At the analysis of each follow‐up point, there were no significant differences in the ASES scores, UCLA scores, and VAS scores between the two groups at the preoperative point and at the 6‐month follow‐up. However, at the 3‐month follow‐up, the ASES scores (59.72 ± 16.43 vs. 66.84 ± 12.82, *p* = 0.003), UCLA scores (22.91 ± 6.12 vs. 24.79 ± 5.46, *p* = 0.045), and VAS scores (3.65 ± 2.59 vs. 2.61 ± 1.91, *p* = 0.005) showed significant differences between the SAC decompression group and the non‐decompression group. The results are displayed in Table 2.

**TABLE 2:** Comparison of patient‐reported outcomes for SAC decompression group vs. non‐decompression group at preoperative point, 3‐month follow‐up, 6‐month follow‐up.

	SAC decompression group (*n* = 68)	Non‐decompression group (*n* = 87)	*p* value	Statistic value
POSS	26 (38.2%)	13 (14.9%)	0.001*	10.997
ASES scores				
T0	50.79 ± 16.19	53.94 ± 15.61	0.222	−1.226
T1	59.72 ± 16.43	66.84 ± 12.82	0.003*	−3.301
T2	77.93 ± 13.09	81.40 ± 12.36	0.092	−1.693
UCLA scores				
T0	20.72 ± 5.83	22.32 ± 5.23	0.074	−1.798
T1	22.91 ± 6.12	24.79 ± 5.46	0.045*	−2.018
T2	29.04 ± 4.23	30.01 ± 4.47	0.173	−1.369
VAS scores				
T0	5.47 ± 1.97	4.99 ± 1.95	0.131	1.519
T1	3.65 ± 2.59	2.61 ± 1.91	0.005*	2.876
T2	1.93 ± 1.83	1.60 ± 1.53	0.225	1.218

Abbreviations: ASES, the American Shoulder and Elbow Surgeons Score; POSS, postoperative shoulder stiffness; SAC, sub‐acromioclavicular; T0, Preoperative point; T1, 3‐month follow‐up; T2, 6‐month follow‐up; UCLA, the University of California–Los Angeles score; VAS, visual analog scale scores.

* Signifies statistical significance: *p* < 0.05.

In the analysis of generalized estimating equations, the results showed a significant difference in the ASES scores [β = −4.971, 95% CI −8.557, −1.278, *p* = 0.008], UCLA scores (β = −1.524, 95% CI −2.798, −0.250, *p* = 0.019), and VAS scores (β = 0.654, 95% CI 0.159, 1.480, *p* = 0.010) throughout the study duration between the SAC decompression group and the non‐decompression group. In addition, there was no interaction effect between group and time in all patient‐reported outcomes scores (Table 3).

**TABLE 3:** Generalized estimating equation analyses of patient‐reported outcomes for SAC decompression group vs. non‐decompression group throughout the study duration.

	β (95% CI)	*p* value	Statistic value
ASES scores			
SAC group vs. non‐decompression group	−4.971 (−8.557, −1.278)	0.008*	7.013
Group*time		0.104	4.520
UCLA scores			
SAC group *vs*. non‐decompression group	−1.524 (−2.798, −0.250)	0.019*	5.500
Group*time		0.507	1.359
VAS scores			
SAC group vs. non‐decompression group	0.654 (0.159, 1.480)	0.010*	6.711
Group*time		0.066	5.434

Abbreviations: ASES, the American Shoulder and Elbow Surgeons Score; CI, confidence interval; SAC, sub‐acromioclavicular; UCLA, the University of California–Los Angeles score; VAS, visual analog scale scores.

* Signifies statistical significance: *p* < 0.05.

## Discussion

### Main Findings

This study found and evaluated the impact of an additional SAC decompression during arthroscopic rotator cuff repair on POSS, which has been noticed in former research.^9^ The main findings of the present study were as follows. First, the SAC decompression is firstly identified as an independent factor for POSS. Second, the mean value of patient‐reported outcomes in the SAC decompression group are significantly lower than non‐decompression group throughout the study duration. This suggests that SAC decompression may increase the likelihood of POSS in comparison with the non‐decompression group (OR = 3.624). Third, there are no difference in patient‐reported outcomes and pain between the two groups in the last follow‐up, indicating that proper treatments can effectively manage POSS.

### Risk Factors for Rotator Cuff Repair Related POSS

Arthroscopic rotator cuff repair is the most frequent surgical procedure for treating rotator cuff tears.^2^, ^3^ Despite its many benefits, one of the most common complications of this surgery is POSS, which can prolong a patient's pain experience and rehabilitation, resulting in higher costs and burdens on medical resources.^14^, ^24^ Previous studies have identified the causes of POSS after surgery, such as scar tissue formation, muscle imbalances, and inactivity.^25^, ^26^, ^27^ The result of binary logistic regression showed that diabetes (OR = 5.464) and SAC decompression (OR = 3.624, 95% CI 1.559–8.427, *p* = 0.003) were independent factors for POSS. Previous research has reported that diabetes increases the risk of POSS, possibly due to the overexpression of serum TGF‐β and TNF‐α in diabetic patients, which exacerbates inflammation and impairs tissue healing.^28^, ^29^, ^30^ This study also provides substantial evidence for the necessity of preoperative risk assessment in diabetic patients. These inflammatory mediators create a localized inflammatory microenvironment that recruits a large number of fibroblasts, which subsequently proliferate and become activated. Upon stimulation by cytokines and growth factors, these activated fibroblasts initiate the PI3K‐AKT signaling pathway, the TGF‐β/Smad signaling pathway, among others. The activation of these pathways drives fibroblasts to produce and secrete large amounts of collagen into the extracellular matrix. As collagen accumulates excessively, it leads to the over‐aggregation of the extracellular matrix, resulting in structural changes to the joint capsule, thereby causing fibrosis and thickening of the shoulder joint.^31^, ^32^, ^33^

### SAC Decompression's Impact on POSS

However, there is limited research to investigate the influence of operative procedures on POSS associated with rotator cuff tears. Sub‐Acromioclavicular Decompression (SAD) is a procedure commonly performed alongside arthroscopic rotator cuff repair, aiming to increase the subacromial space for the supraspinatus tendon while removing bone spurs, osteophytes, and excessive synovial tissue from the inferior surface of the acromioclavicular joint.^19^, ^34^ Cai's measurements of shoulder morphology parameters revealed that reducing osteophytes and bone growth under the acromioclavicular joint enlarges the subacromial space, preventing further damage to the supraspinatus tendon.^35^ Snyder and Jaeger found that performing SAD concurrently with arthroscopic rotator cuff repair reduces the risk of re‐tearing and improves surgical outcomes. However, Khorshad reported that SAD might exacerbate shoulder pain, potentially necessitating additional treatments to alleviate discomfort.^21^, ^34^, ^36^

In clinical practice, the impact of SAD on POSS is a controversial and unresolved issue, requiring surgeons to carefully weigh the risks and benefits when making surgical decisions.^20^, ^37^ This study found that SAD independently increases the risk of POSS compared with the control group, possibly due to increased soft tissue damage and inflammation. Extensive synovial tissue resection and capsular injury during SAD may alter the shoulder's normal biomechanics, leading to excessive scarring and adhesions.^14^, ^34^, ^38^ Moreover, SAD involves greater surgical trauma, which increases the release of inflammatory mediators and exacerbates the postoperative inflammatory response. Although inflammation is a natural part of the healing process, excessive inflammation can lead to scar tissue formation and prolonged healing, ultimately restricting shoulder mobility and worsening postoperative stiffness.^24^, ^39^ To gain a more comprehensive understanding of this impact, future research should establish animal models to explore the specific mechanisms of SAD surgery and assess its long‐term effects through extended follow‐up.

It is important to note that there are many other clinical factors that can contribute to the development of POSS, including age, sex, diabetes, the grade of muscle fatty infiltration, the size and location of the tears, the type of surgery, and the rehabilitation protocol.^14^, ^17^, ^40^ Additionally, some studies have shown that the addition of SAC decompression was performed in surgery can result in positive clinical outcomes with long‐term follow‐up, especially in those with partial‐thickness tears (90.9%) or full‐thickness tears (70.6%).^21^ Given the various risk factors, orthopedic surgeons must make informed decisions on whether to perform SAC decompression based on the individual needs and goals of their patients. Orthopedic surgeons should carefully weigh the potential benefits and risks of SAC decompression and discuss the operative procedures with their patients to determine the best course of action.

In this study, the diagnosis of POSS was treated promptly, and patients were encouraged to collaborate with their doctors to develop a personalized rehabilitation program that would help minimize the harm of POSS. Physical therapy, massage therapy, and other treatments can help to reduce scarring and adhesions in the joint, enhance ROM, and restore shoulder strength and function.^34^, ^41^, ^42^ To make a more appropriate choice of rehabilitation plan, further randomized controlled trials are necessary to understand the clear influence of SAC decompression on POSS and animal experiments are need to clarify the pathogenic mechanism of POSS after SAC decompression.

### Limitations and Prospect

First, the small sample size and lack of a randomized controlled trial reduced the generalizability of the findings to the larger population and more multicenter randomized controlled trials are needed to confirm these results. Additionally, the limited follow‐up time in this study made it difficult to assess the long‐term outcomes of the procedure and its impact on rehabilitation. Second, although several clinical factors were considered in the study, there may be other factors that were not controlled for and could affect the results. Finally, this study showed that SAC decompression increases the risk of POSS, but this does not imply that it is not beneficial in treating rotator cuff tears. Orthopedic surgeons should carefully weigh the potential benefits and risks of SAC decompression for each individual patient.

## Conclusions

In conclusion, SAC decompression increases the risk of POSS compared with the non‐decompression group after arthroscopic rotator cuff repair, which indicate surgeons do not perform SAC decompression unless necessary.

## Author Contributions

CL designed and initiated the trial. ZLW and MIA were responsible for logistics, patient recruitment, and data collection. RY was responsible for the operating and leading. YA, MZ, and YL carried out experimental design, statistical analysis plan, and writing of the manuscript. CL and ZLW drafted the manuscript. And all the authors read and approved the final manuscript.

## Conflict of Interest Statement

The authors declare no conflicts of interests.

## Ethics statement

This registry‐based study was approved by the ethics committee of the Sun Yat‐sen memorial hospital (SYSKY‐2023‐449‐01).

## Funding Information

This study was supported by the National Natural Science Foundation of China (nos. 81972067 and 82002342) and the Fundamental Research Funds for the Central Universities, Sun Yat‐sen University (no. 2020004).
